# Disturbance of cellular homeostasis as a molecular risk evaluation of human endothelial cells exposed to nanoparticles

**DOI:** 10.1038/s41598-021-83291-0

**Published:** 2021-02-15

**Authors:** Paulina Wigner, Krzysztof Zielinski, Sylwia Michlewska, Paulina Danielska, Agnieszka Marczak, Eduardo Junior Ricci, Ralph Santos-Oliveira, Marzena Szwed

**Affiliations:** 1 Department of Medical Biophysics, Institute of Biophysics, Faculty of Biology and Environmental Protection, University of Lodz, Lodz, Poland; 2 Laboratory of Microscopic Imaging and Specialized Biological Techniques, Faculty of Biology and Environmental Protection, University of Lodz, Lodz, Poland; 3 Laboratory of Nanodrugs, Faculty of Pharmacy, Rio de Janeiro Federal University, Rio de Janeiro, Brazil; 4 Laboratory of Nanoradiopharmaceuticals, Research Group of Nano-Radiopharmaceuticals and Novel Radiopharmaceuticals, Zona Oste State University, Rio de Janeiro, Brazil; 5 Laboratory of Nanoradiopharmacy and Synthesis of Novel Radiopharmaceuticals, Nuclear Engineering Institute, Brazilian Nuclear Energy Commission, Rio de Janeiro, Brazil

**Keywords:** Biochemistry, Biotechnology, Cell biology, Chemical biology

## Abstract

Even though application of nanoparticles in medicine seems to provide unique solutions for drug delivery and diagnosis diseases, understanding interactions between nanoscale materials and biological systems is imperative. Therefore, this study determined the effect of different types of nanoparticles (NPs) on human endothelial cells and examined the types of toxicity responses they can induce. Four different types of NPs were tested (PLA/MMT/TRASTUZUMAB, PLA/EDTMP, PLGA/MDP, and Pluronic F127 MICELLES), representing three putative areas of application: anticancer therapy, scintigraphy, and cosmetology. The experiments were performed on immortalized human umbilical vein endothelial cells (HUVEC-STs). Light contrast phase microscopy as well as cell viability assays showed that only Pluronic F127 MICELLES decreased the number of HUVEC-STs in contrast to PLA/MMT/TRASTUZUMAB, PLA/EDTMP, and PLGA/MDP NPs, which altered cell morphology, but not their confluency. The tested NPs induced not only DNA strand-breaks and alkali-labile sites, but also internucleosomal DNA fragmentation, visualized as a DNA ladder pattern typical of apoptosis. Moreover, generation of free radicals and subsequent mitochondrial membrane potential collapse showed the significance of free radical production during interactions between NPs and endothelial cells. High concentrations of NPs had different degrees of toxicity in human endothelial cells and affected cell proliferation, redox homeostasis, and triggered mitochondrial dysfunction.

## Introduction

Nanoparticles (NPs) can potentially be used in many different fields of science^1^. As submicroscopic structures (1–500 nm), they are used in medicine (as potential transporters of drugs in targeted therapy and imaging), engineering (production of nanocomposites), cosmetology (mineral UV filters in cosmetics), or pharmaceutical products increasing circulation and microcirculation. The most characteristic NP properties are their small size, large surface area to mass ratio, and high reactivity. On the other hand, the ultra-small NP size and their unique properties allow them to be used as oral, inhalational, dermal, or intravenous nanodrugs. Consequently, they can be transported to several organs and tissues to reach blood vessels^2^.

Vascular endothelium is a dynamic tissue that covers human blood vessel lumen. The capacity to synthesize proteins associated with basal lamina and matrix metalloproteinases makes endothelial cells (ECs) an important factor in the vascular remodelling process^3^. Due to phenotypic heterogeneity and the presence of tight junctions, the endothelium regulates solute flux and fluid permeability between the blood and other tissues. Moreover, ECs have significant autocrine, paracrine, and endocrine actions, influence smooth muscle cells and platelets, and contribute to important angiogenesis processes^4^. Specific transport mechanisms are involved in blood circulation of macromolecules to the underlying interstitium and cells. Many substances (drugs, antibodies, and hormones) interact with the vessel wall and modulate both the metabolism and function of the endothelial barrier^5^. For this reason, the endothelium is extremely important in nanotoxicological studies, mainly due to the possible interactions between ECs and nanosubstances. In addition, the use of NPs can modulate endothelial leakiness and have been increasingly employed in recent studies to overcome vascular barriers, improve drug delivery, and enhance cellular uptake of nanodrugs^6^.

The influence of nanosubstances on the endothelial function is a key factor for better understanding of the potential in vivo NP effects^7^. It is a huge challenge to find the best cellular model for the evaluation of nanomaterial effect on the human blood vessel lumen. There are many variables that should be considered when human umbilical vein endothelial cells (HUVECs) are used in an in vitro model to assess NP toxicity. Consequently, NP physicochemical properties (size, shape, solubility, and surface charge) define the diversity of EC viability and proliferation as molecular responses to NP exposure^8^. For example, the polyvalent surface of NPs may induce cross-linking of cellular receptors, initiate signaling processes, induce structural alterations at the cell surface, and interfere with normal endothelial function^9^. Moreover, the physicochemical properties of nanosubstances can cause undesirable toxic effects associated with long-term application of NPs.

HUVECs were developed via fusion of primary cell cultures with permanent cell lines. Gene and protein profile analysis has indicated that immortalized cells often retain most of the important endothelial markers^10,11^. However, there are other cellular models that are crucial for understanding the EC reaction to nanomaterials application. For instance, human aortic endothelial cells (HAECs) are a good choice for in vitro cell culture if the research is targeted on restenosis after vascular intervention triggered by NPs^12^. Some data have described a more complicated system with microfluidic channels lined with hCMEC/D3 cells, constructed to examine the effect of NP shape on particle adhesion^13^. On the other hand, computational fluid dynamics (CFD) is a well-known in silico approach to find the connection between NP properties and their ability to modulate immune system interactions and blood clearance profile^14^.

**Figure 1:**
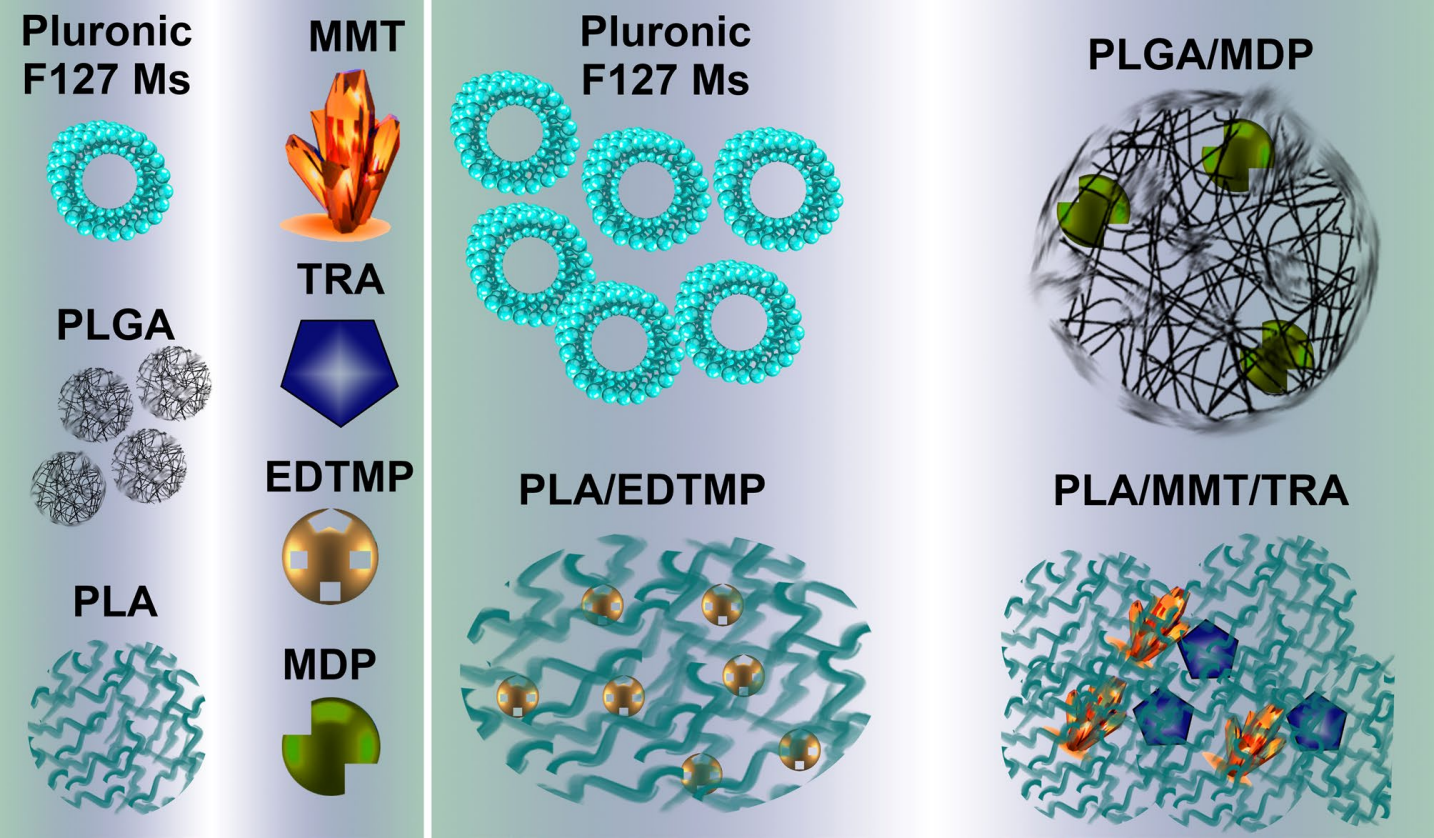
The schematic overview of the four nanoparticles used in the study.

The present study aimed to verify the hypothesis that NPs can reveal cytotoxic properties and modulate endothelial cell homeostasis even though they do not always reduce cell viability. Therefore, this basic and cognitive study investigated different types of molecular responses induced by endothelial NP interactions. Four types of NPs were used (Table 1, Fig. 1): the monoclonal antibody, TRASTUZUMAB (PLA/ MMT/TRA) modified with polylactic acetate (PLA) and montmorillonite (MMT), which specifically targets HER2 receptor extracellular domain^15^; ethylene diamine N,N,N',N' tetramethylene phosphonic acid NPs coated with PLA (PLA/EDTMP)^16^, which are used in breast and bone cancer treatment; silicon NPs PLGA/MDP modified with methylene diphosphate (MDP) and coupled to poly lactic-co-glycolic acid (PLGA)^17^, which can be used in scintigraphy; and Pluronic F127 MICELLES (Pluronic F127 Ms), that can be administered dermally^18^, intranasally, or intravenously^19^. HUVEC-STs were used as a cellular model. Thus, multi-dimensional analyses were performed to evaluate interactions between NPs and ECs. Phase contrast microscopy demonstrated that NPs altered the morphology of HUVEC-STs. NPs also induced DNA damage that did not always correspond to inhibition of cellular proliferation and EC viability. The cytotoxic effect of the investigated nanosubstances was studied for up to 72 h with particular emphasis on the induction of programmed cell death, including reactive oxygen species (ROS) production. Mitochondrial stress monitoring was used to determine the importance of redox homeostasis disturbances in ECs triggered by NP interactions.

**Table 1:** Physicochemical properties of investigated particles: size distribution, zeta-potential, PDI, and entrapment efficacy of PLA/MMT/TRA, PLA/EDTMP, PLGA/MDP, and Pluronic F127 Ms.

Nanoparticles	Application	NP size^a^ (nm)	ζ potential (meV)	PDI	Entrapment efficacy (% of drug)
PLA/MMT/TRA	Monoclonal antibodies against HER-2 receptors, used in breast cancer treatment, high cardiotoxicity	278 ± 4.2	0.4 ± 0.6	0.21	90
PLA /EDTMP	Part of technetium izotope 99mTc complex, used in scintigraphy for bone fracture research	265.9 ± 2.8	0.2 ± 0.1	0.19	70
PLGA/MDP	Component of Quadramet medicament used in bone cancer treatment, very toxic to bone marrow	207 ± 2.4	4.6 ± 1.5	0.27	78
Pluronic F127 ms	Applied as basis components for cosmetics and dermatology drugs, mostly to increase UV protection	140 ± 1.2	7.9 ± 0.2	0.3	100

^a^The size from each nanosystem has been confirmed by TEM or AFM analysis and the results were similar with the DLS analysis.

## Results

### Physiochemical characterization of NPs

The examined NPs had a hydrodynamic diameter ranging from 140 to 278 nm with a near neutral or low positive charge, a zeta potential of + 0.4 to + 7.9 mV (Table 1), and polydispersity index (PDI) varying from 0.19 to 0.30. The PDI values confirmed the medium dispersity of all NPs. NP entrapment efficacy varied from 70% (PLA/MDP) to 100% (Pluronic F127 Ms), supporting the efficient encapsulation of all drugs used during the NP preparation (Table 1). The NP size was stable upon incubation in PBS when stored at room temperature for more than 24 h (data not shown).

### Various sensitivities of human ECs to NP treatment

**Figure 2:**
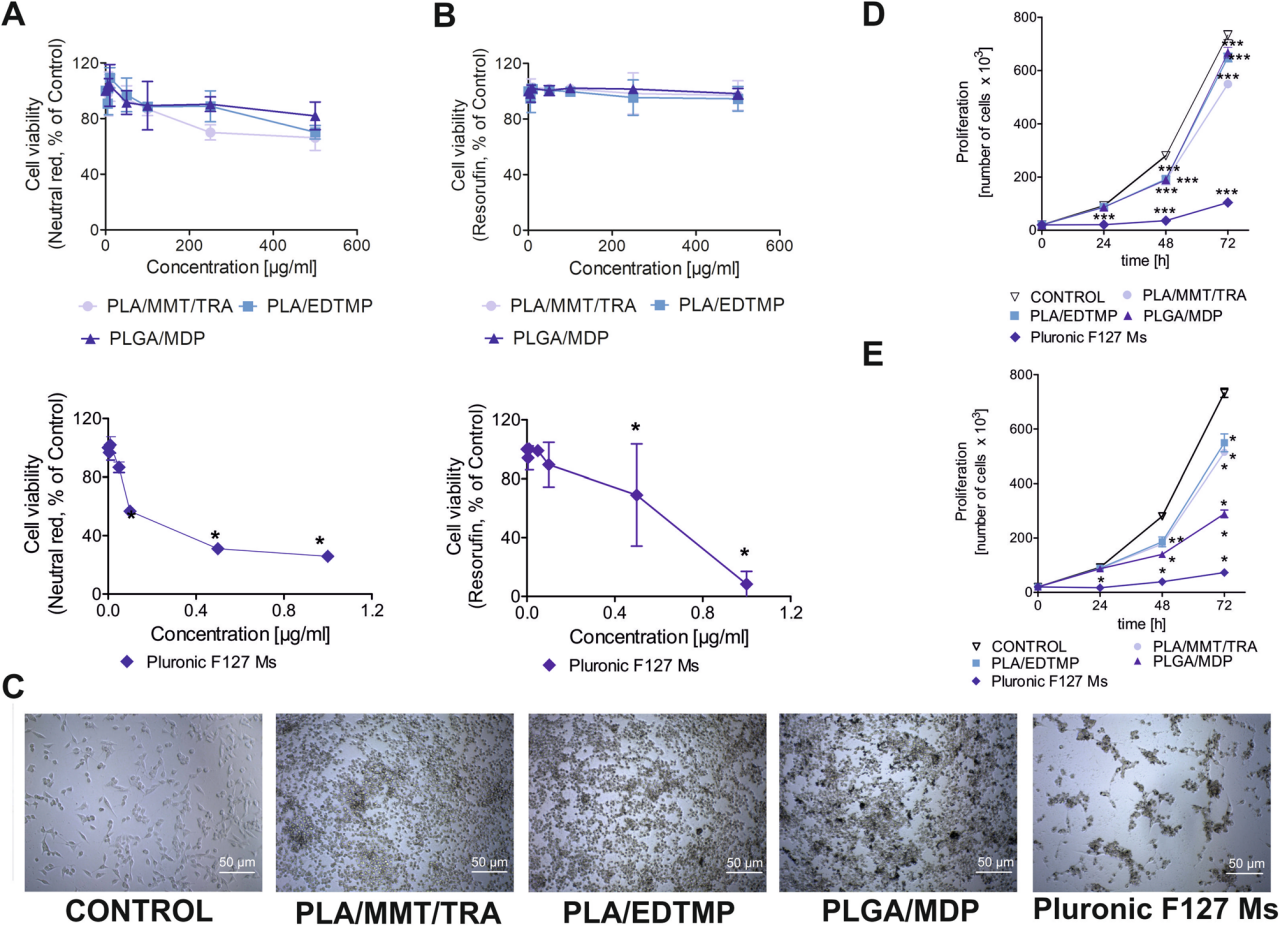
Comparisons of NP cytotoxicity in human endothelial cell line. (**A**,**B**) HUVEC-ST cell viability measured by the neutral red assay (left panels) or Alamar Blue assay (resazurin oxidation; right panels) after cell incubation for 72 h with increasing concentrations of NPs. Values are the mean ± SD of three independent experiments with six replicates in each experiment. Statistically significant differences comparing control cells taken as 100%, *p < 0.05 (**C**) Light microscopy images under contrast phase conditions of HUVEC-STs after treatment for 24 h with 100 μg/mL of PLA/MMT/TRA, PLA/EDTMP, PLGA/MDP, or 0.025 μg/mL of Pluronic F127 Ms. Bar = 50 µm. (**D**,**E**) Growth kinetics of HUVEC-STs treated with PLA/MMT/TRA, PLA/EDTMP, PLGA/MDP [100 μg/mL (**D**) or 250 μg/mL (**E**)], or Pluronic F127 Ms [0.25 μg/mL (**D**) or 0.5 μg/mL (**E**) for 72 h. ***p < 0.001, values are % of those obtained for untreated control (mean ± SD; n ≥ 3).

When particle cytotoxicity was compared by adding NPs at increasing concentrations to HUVEC-STs, a striking difference in NP cytotoxicity was observed between the four types of NPs. Data obtained with both neutral red assay and resazurin measurements (Alamar Blue test) showed the various NP cytotoxicity values following incubation for 72 h. For instance, Pluronic F127 Ms showed the highest cytotoxicity, whereas PLA/MMT/TRA, PLA/EDTMP, and PLGA/MDP NPs were the least toxic (Fig. 2A,B). This is in agreement with our previous analyses of NP cytotoxicity in peripheral blood mononuclear cells^20^. The morphological changes of human ECs induced by 24-h exposure to 100 μg/mL of PLA/EDTMP, PLGA/MDP, and PLA/MMT/TRA or 0.025 μg/mL of Pluronic F127 Ms were visualized in parallel. The microscopy images showed that Pluronic F127 Ms had the highest toxic effect (cell detachment), whereas PLA/MMT/TRA, PLA/EDTMP, and PLGA/MDP at this time point mostly led to cell rounding and did not reduce cell numbers (Fig. 2C). Cell proliferation rate is a measurement of cell sensitivity to various types of external factors. Therefore, it was determined whether the examined NPs had a toxic effect on HUVEC-ST proliferation by counting the cells after trypan blue staining for up to 72 h. A reduction in cell number was observed after HUVEC-STs were treated with Pluronic F127 Ms (Fig. 2D). A severe reduction in cell proliferation when HUVEC-STs were incubated with PLA/MMT/TRA, PLA/EDTMP, and PLGA/MDP for up to 24 h was not observed. It should be noted that a significant reduction was observed, although with a slightly different extent and kinetics, when HUVEC-STs were incubated with a higher concentration of PLA/MMT/TRA, PLA/EDTMP, and PLGA/MDP (250 μg/mL) for up to 72 h (Fig. 2E).

### Genotoxic effect of NPs

**Figure 3:**
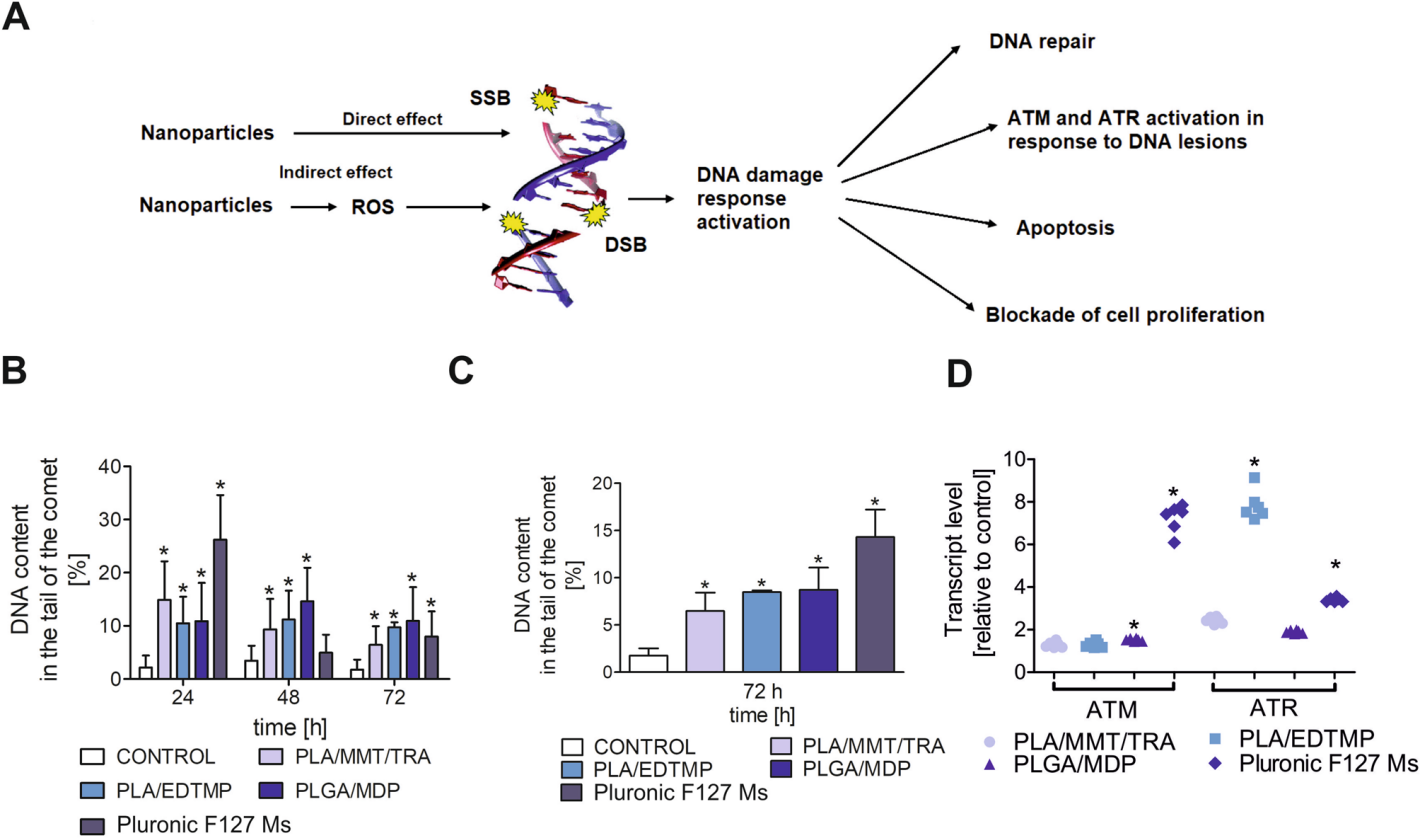
Genotoxic NP activity in human endothelial cells (**A**) Schematic overview of DNA damage and its consequences. (**B**,**C**) NP genotoxicity in HUVEC-STs. Cells were treated with PLA/MMT/TRA, PLA/EDTMP, PLGA/MDP (100 μg/mL), or Pluronic F127 Ms (0.25 μg/mL) (**B**) for up to 72 h or incubated only for 72 h with double doses of NPs: PLA/MMT/TRA, PLA/EDTMP, PLGA/MDP (200 μg/mL), or Pluronic F127 Ms (0.5 μg/mL) (**C**). DNA damage was evaluated by measuring DNA percentage in comet tail by alkaline version of comet assay (pH > 13). *p < 0.05, significant differences between NP-treated and control cells. Fifty images were analysed from each slide. Data are the means ± SD of three independent experiments. (**D**): ATM and ATR gene transcript expression (relative to HPRT1 housekeeping gene) in HUVEC-STs exposed to NPs for 24 h. Bars represent the means ± SD of three separate experiments (n 6). Asterisks refer to level of significant (*p < 0.05, n = 6) difference in cell transcription treated with investigated NPs compared to untreated cells.

A reduction in proliferation rate (Fig. 2D,E) in cell cultures is often a hallmark of altered cellular homeostasis when DNA damage is induced^21^. Therefore, the study evaluated whether the investigated NPs triggered DNA single- and double-strand breaks as well as alkali-labile sites on the basis of degradable DNA percentage. When HUVEC-STs were treated with Pluronic F127 Ms for up to 24 h (Fig. 3B), an increase in DNA percentage in the comet tail was noted. Moreover, significant differences between the extent of DNA damage induced by PLA/EDTMP, PLGA/MDP, and PLA/MMT/TRA were detected 24, 48, and 72 h after exposure of ECs to 100 μg/mL of NPs. On the other hand, a nearly two-fold decrease in DNA percentage in the comet tails (Fig. 3C) was noted in cells treated with Pluronic F127 Ms if incubation continued for up to 72 h, suggesting that HUVEC-STs needed a longer time for DNA repair machinery to be activated as a consequence of cellular stress initiation by NPs.

Because human ECs were able to cope with the accumulation of DNA damage induced by PLA/EDTMP, PLGA/MDP, and PLA/MMT/TRA, it was necessary to determine how early the genes involved in a response to one or more DNA lesions could be activated. Human cells contain highly conserved pathways to signal and repair DNA damage during constant exposure to exogenous and endogenous DNA-damaging agents (Fig. 3A). ATM and ATR are near the top of these signalling networks, which are often named the “sentries” at the gate of genome stability. Therefore, the present study evaluated whether the expression of these proteins can be induced in human ECs by treatment with the tested NPs. ATM and ATR kinase transcription was increased when the cells were treated with Pluronic F127 Ms for up to 24 h (Fig. 3D). Neither PLGA/MDP nor PLA/MMT/TRA triggered extensive transcription of ATR or ATM mRNA. However, when HUVEC-STs were treated with PLA/EDTP, a seven-fold increase in the transcription of ATR kinase was observed. This also suggested that a different set of DNA lesions was induced in HUVEC-STs, as it is known that ATR (different to ATM) is mainly activated by single-strand DNA breaks^22^.

### Internucleosomal DNA fragmentation of ECs is part of DNA lesions caused by NPs

**Figure 4:**
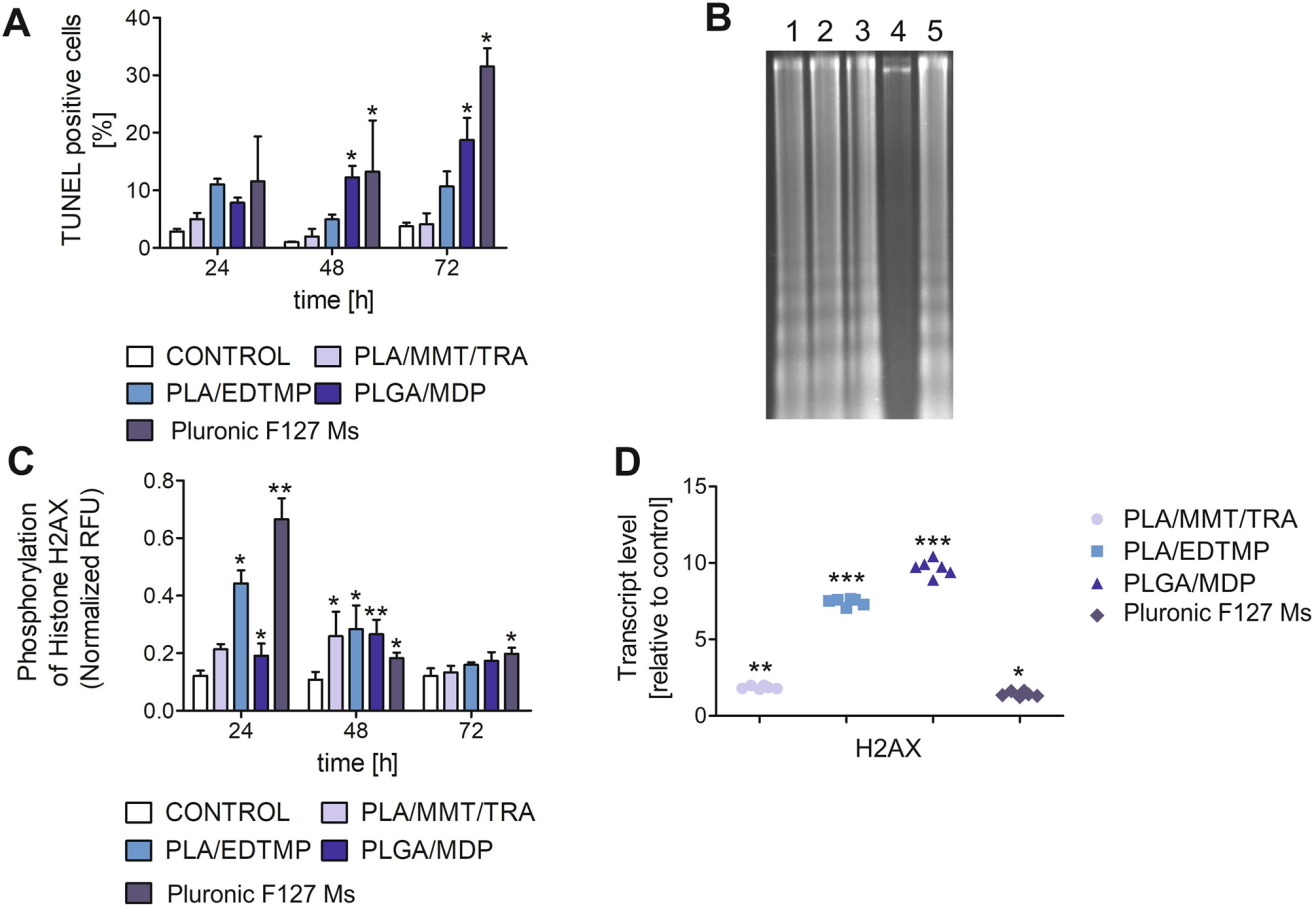
DNA damage in HUVEC-STs triggered by NPs is the result of apoptosis. (**A**) Induction of apoptosis-dependent DNA fragmentation in HUVEC-STs after treatment with NPs [PLA/MMT/TRA, PLA/EDTMP, PLGA/MDP (100 μg/mL), or Pluronic F127 Ms (0.25 μg/mL)] at 24, 48, and 72 h estimated by the TUNEL assay. Results represent the mean ± SD of three independent experiments. *p < 0.05 for significant differences between NP-treated and control cells. (**B**) DNA fragmentation induced by NPs detected by agarose gel electrophoresis of DNA isolated from human endothelial cells. HUVEC-STs were treated for up to 72 h with 100 μg/mL of PLA/EDTMP, PLGA/MDP, and PLA/MMT/TRA or 0.025 μg/mL of Pluronic F127 Ms; 1—PLA/MMT/TRA, 2—PLA/EDTMP, 3—PLGA/MDP, 4—Control, 5—Pluronic F127 Ms. (**C**) NP effect (100 μg/mL of PLA/EDTMP, PLGA/MDP, and PLA/MMT/TRA or 0.025 μg/mL of Pluronic F127 Ms) on histone H2AX phosphorylation in human endothelial cells. *p < 0.05, **p < 0.01 for significant differences between NP-treated and control cells. Data are the means ± SD of three independent experiments. (**D**) Level of mRNA transcripts for histone H2AX gene in HUVEC-STs exposed to NPs (100 μg/mL of PLA/EDTMP, PLGA/MDP, and PLA/MMT/TRA or 0.025 μg/mL of Pluronic F127 Ms) for 24 h. Data are expressed as the means ± SD, (n = 3). Asterisks refer to level of significant (*p < 0.05, ***p < 0.001) difference in mRNA level in NP-treated cells compared to untreated control cells.

As described above, HUVEC-STs have been shown to be vulnerable to genotoxic effects of the investigated NPs. As the comet assay has been broadened to include the detection of DNA fragmentation in cells undergoing apoptosis^23^, it was first confirmed whether apoptosis-dependent DNA fragmentation was induced by the investigated NPs. Indeed, the population of TUNEL-positive cells (those that exhibited single- and double-stranded DNA fragments with possible label-free 3′ OH ends) increased in a time-dependent manner with all tested NPs (Fig. 4). The maximum increase in the percentage of apoptotic cells with DNA strand-breaks was detected 72 h after PLGA/MDP and Pluronic F127 Ms treatment. These data correlated well with oligosomal DNA fragmentation monitored during the DNA ladder assay. The characteristic DNA ladder-like pattern of the internucleosomal DNA cleavage (Fig. 4B, Supplementary Fig. S1) was detected when HUVEC-STs were incubated for up to 72 h with all tested NPs (100 μg/mL of PLA/EDTMP, PLGA/MDP, and PLA/MMT/TRA or 0.025 μg/mL of Pluronic F127 Ms).

Cellular stress response proteins are activated when DNA damage is initiated by various stressful conditions. One of these response proteins downstream of apoptosis-induced DNA damage is the phosphorylation of histone H2AX. PLA/EDTMP, PLGA/MDP, and Pluronic F127 Ms triggered histone H2AX phosphorylation after just 24 h of NP incubation (Fig. 4C). On the other hand, a statistically significant effect of all NPs was observed when HUVEC-STs were exposed to NPs for up to 48 h. Interestingly, and in support of this, NP treatment led to an increase in histone H2AX transcription (Fig. 4D), as the first, cellular response to DNA lesions.

### Apoptosis induction in HUVEC-STs after NP treatment

**Figure 5:**
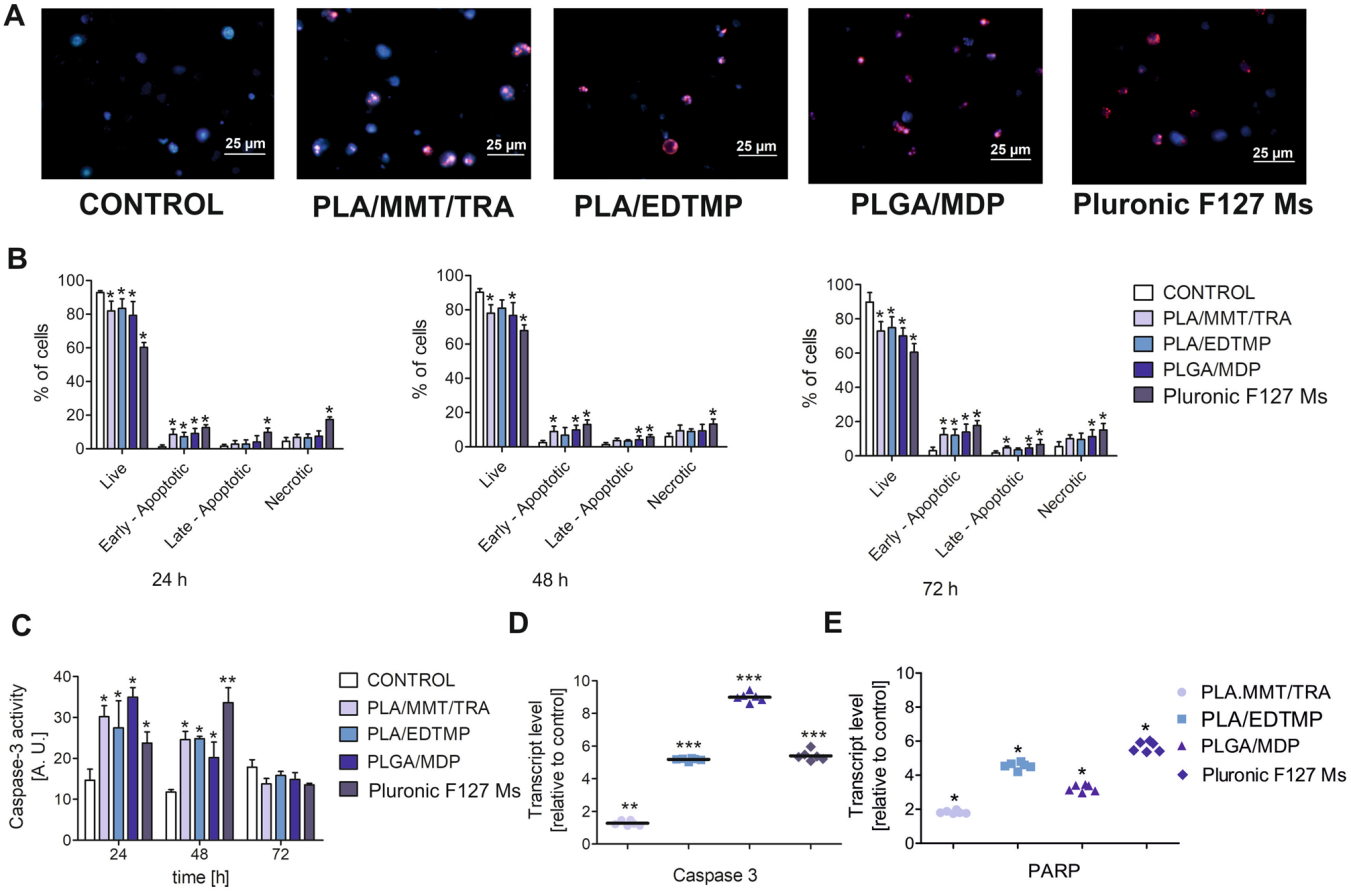
Molecular markers of NP-induced apoptosis in human endothelial cells. (**A**) Double staining with DNA-specific dye (Hoechst 33258 and PI) of HUVEC-STs after NP treatment (100 μg/mL of PLA/EDTMP, PLGA/MDP, and PLA/MMT/TRA or 0.025 μg/mL of Pluronic F127 Ms) for 72 h. Cells were analysed under fluorescence microscope (Olympus IX70, Japan; magnification: 400 ×). (**B**): Quantification of live, early apoptotic, late apoptotic, and necrotic HUVEC-STs at different times following treatment with NPs in the same conditions as described above. Cells were stained with a mixture of Hoechst 33258/PI. Representative data are shown from three separate experiments. p < 0.05 for significant differences between NP-treated and control cells (**C**) Time-dependent changes in caspase-3 activity in HUVEC-STs treated with various concentrations of NPs (100 μg/mL of PLA/EDTMP, PLGA/MDP, and PLA/MMT/TRA or 0.025 μg/mL of Pluronic F127 Ms) for 24, 48, and 72 h. Results represent the means ± SD of three independent experiments. *p < 0.05 for significant differences between NP-treated and untreated control cells. (**D**) Caspase-3 and PARP transcription levels (relative to HPRT1) in HUVEC-STs exposed to examined nanosubstances: PLA/EDTMP, PLGA/MDP, and PLA/MMT/TRA (100 μg/mL) or Pluronic F127 Ms (0.025 μg/mL) for 24 h. Asterisks refer to significant differences (*p < 0.05, **p < 0.01, and **p < 0.001; n = 3) in transcription levels in NP-treated cells compared to untreated cells.

Intrigued by the possibility of DNA fragmentation caused by different cytotoxic effects of NPs, the present study aimed to identify the fraction of apoptotic and necrotic dead cells (Fig. 5A) using a mixture of two fluorochromes Hoechst 33258/PI. The four cell populations: live, early apoptotic, late apoptotic, and necrotic were distinguished during fluorescence microscopy observations (Fig. 5B). A time-dependent difference between the investigated NPs was not detected. It appeared that HUVEC-STs exhibited the same resistance pattern to NPs. Slight differences between the NPs were noted when incubation was prolonged for up to 72 h. These data are in agreement with cytotoxicity studies performed using the neutral red and Alamar Blue assays. Apoptosis induction was further detected via fluorogenic CellEvent caspase-3 substrate staining. The highest caspase-3 activity we detected after 24 and 48 h of treatment with all investigated NPs (Fig. 5C). In contrast to PLA/MMT/TRA, PLA/EDTMP, and PLGA/MDP, the maximal cleavage of caspase-3 substrate was observed when cells were incubated with Pluronic F127 Ms for up to 48 h. These data are in agreement with caspase-3 mRNA transcription measured after 24 h (Fig. 5D). It should be noted that the expression, as well as transcription, was the highest in PLGA/MDP-treated samples. As an increase in PARP functionality may be a consequence of alterations in caspase-3 activity, PARP mRNA level was measured after 24 h of incubation with NPs. All investigated NPs altered mRNA PARP transcription, which was directly related to the initiation of DNA repair pathways (Fig. 5E).

### Mitochondrial stress is triggered in ECs via NP properties

**Figure 6:**
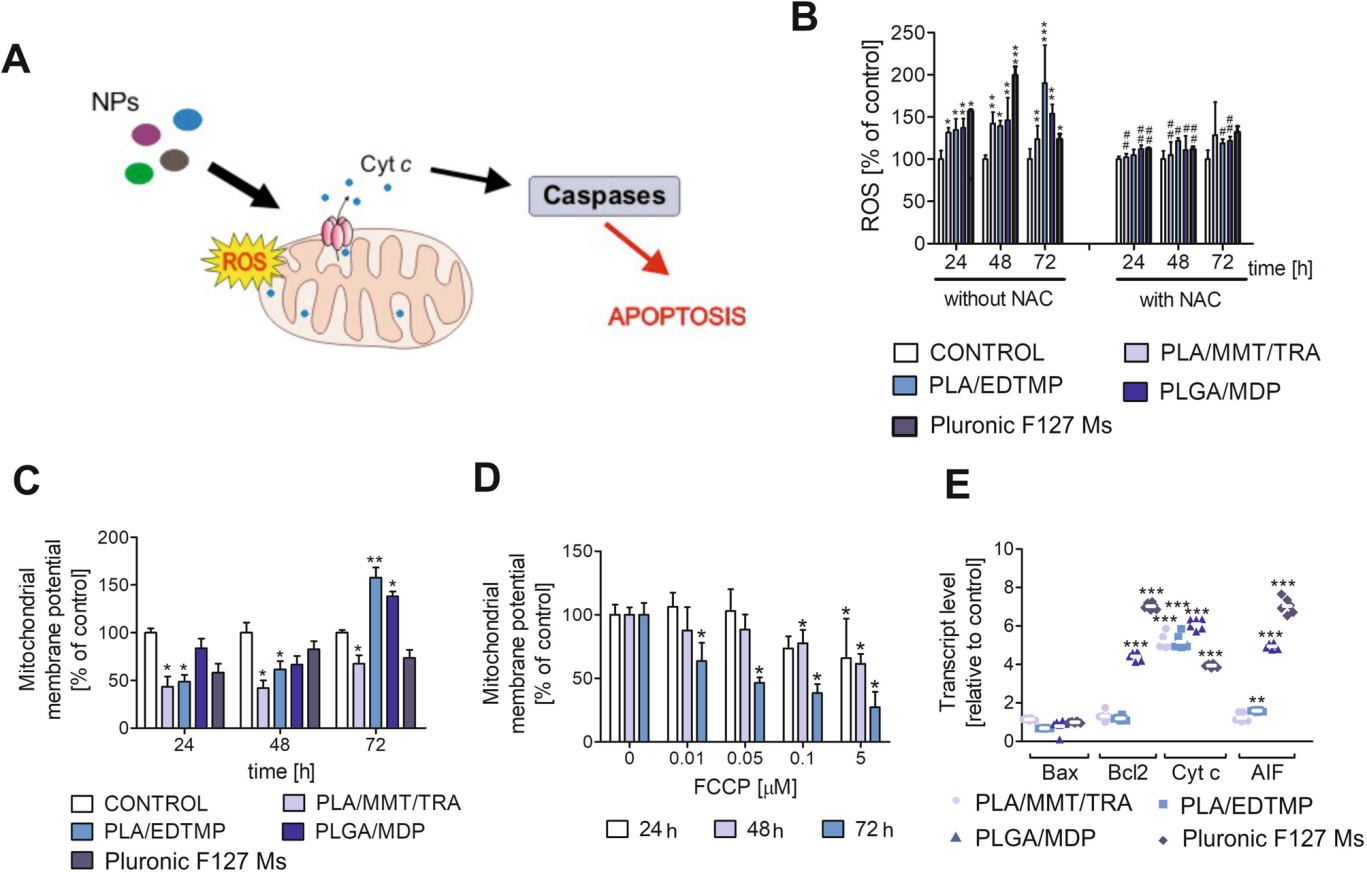
Mitochondrial stress triggered by NPs in HUVEC-STs. (**A**) Schematic overview of perturbations in mitochondrial functions, which may occur after NP treatment. (**B**) ROS production in cells treated with NPs (100 μg/mL of PLA/EDTMP, PLGA/MDP, and PLA/MMT/TRA or 0.025 μg/mL of Pluronic F127 Ms) for 24, 48, and 72 h. DCF fluorescence intensity in control cells was set to 100%. Each value represents average ± SD of four independent experiments, *p < 0.05, **p < 0.01, and ***p < 0.001 for significant differences compared to control cells, ^#^p < 0.05, ^##^p < 0.01 for significant changes compared to samples preincubated with NAC and subsequently incubated with nanosubstances. (**C**,**D**): Changes in mitochondrial membrane potential of HUVEC-STs seeded into black 96-well titration microplates and incubated with NPs (100 μg/mL of PLA/EDTMP, PLGA/MDP, and PLA/MMT/TRA or 0.025 μg/mL of Pluronic F127 Ms) for 24, 48, and 72 h (**C**) or FCCP (**D**). Fluorescence ratio of JC-1 dimers to JC-1 monomers in control was assumed to be 100%. Results are presented as means ± SD of four experiments. *p < 0.05, **p < 0.01 for significant differences between NP-treated and untreated control cells (taken as 100%); (**E**) Bax, Bcl2, Cyt c, and AIF gene transcript expression (relative to HPRT1 housekeeping gene) in HUVEC-STs exposed to examined nanosubstances: PLA/EDTMP, PLGA/MDP, and PLA/MMT/TRA (100 μg/mL) or Pluronic F127 Ms (0.025 μg/mL). Asterisks refer to level of significant (**p < 0.01, ***p < 0.001; n = 3) difference in expression in cells treated with investigated NPs compared to untreated cells.

ROS generation related to the oxidation chain mitochondrial metabolism is one of the markers confirming apoptosis induction (Fig. 6A). Increased free radical production was observed in NP-treated cells as a consequence of oxidative stress generation. ROS production was considerably greater in cells incubated with PLA/EDTMP, PLGA/MDP, and Pluronic F127 Ms, reaching a maximal level after 48 h of incubation (Fig. 6B). At this time point, a two-fold and 1.7-fold increase was noted in cells incubated with PLA/EDTMP and PLGA/MDP or Pluronic F127 Ms, respectively. On the other hand, the highest level of ROS production in HUVEC-STs treated with PLA/MMT/TRA was noted after 72 h of treatment and increased by approximately three-fold in comparison to untreated control cells. Almost complete inhibition of ROS production was observed in cells pretreated with N-acetyl cysteine (NAC).

It has been previously described that the role of mitochondria in programmed cell death induction is related to the changes in mitochondrial membrane potential (MMP, Δψ_m_)^24^. Early MMP depletion was measured using JC-1-stained cells in a fluorescence microplate reader assay. FCCP was used as a positive control (Fig. 6D), which is an ionophore that disrupts ATP synthesis. The collapse of Δψ_m_ in human ECs was observed after 24 and 48 h of treatment with PLA/MMT/TRA, PLA/EDTMP, and PLGA/MDP (Fig. 6C). Interestingly, an increase in JC-1 fluorescence was observed when incubation with these three NPs was prolonged for up to 72 h. At this time point, MMP increased by about 50% when HUVEC-STs were cultured with PLA/EDTMP and PLGA/MDP, which may suggest hyperpolarization of the inner mitochondrial membrane.

The Δψ_m_ changes are often one of the factors that can confirm mitochondrial homeostasis disturbances. Indeed, during mitochondrial stress, many molecules are imported from the intermembrane space or matrix to the cytosol. Therefore, it was determined whether the mRNA level of Bax, Bcl-2, cytochrome c (Cyt c), and AIF changed as a consequence of transcription alteration of these genes. Neither Bcl-2 nor Bax transcription was changed after incubation with NPs for up to 24 h, apart from Pluronic F127 Ms, which triggered a 4.5-fold increase in Bcl-2 transcription (Fig. 6E). However, a significant increase was noted for Cyt c and AIF mRNA under the same treatment conditions, when all NPs induced an increase in gene transcription of at least two-fold, indicating that apoptosis was induced before cell rupture.

## Discussion

As NPs can easily penetrate into the body, they can be transported to various organs or tissues and affect them in many different ways. There are many chemical modifications used during the synthesis of well-known NPs that increase their circulation time, limit aggregation, or decrease their association with untargeted serum and tissue proteins^25^. However, several reports have indicated that the strategies used to coat NPs may negatively influence the performance of NPs as drug carriers^26,27^. Human blood vessels are one of the first barriers that NPs interact with. Thus, it is necessary to understand the adverse effects of NPs on human ECs that cover the lumen of arteries, veins, capillaries, and venules.

In the present study, four different types of NPs were used, including PLA/EDTMP, PLGA/MDP, PLA/MMT/TRA, and Pluronic F127 Ms, to study cellular responses and human EC sensitivity to various nanomaterials. It is worth mentioning that both nanomaterials and their components can have cytotoxic properties during the evaluation of NP toxicity. For example, the major side effects of ^153^Sm-EDTMP NPs are a reduction of platelets and leucocytes in peripheral blood caused by β-particles from the radiopharmaceutical attached to the bone matrix^16^.

In the present study, Pluronic F127 Ms were the most toxic after 72 h of incubation, as determined using both resazurin oxidation and neutral red assays. However, the effect was greater during the assessment of neutral red accumulation inside the lysosomes. This may suggest that Pluronic F127 Ms cytotoxicity is related to a decrease in lysosome functionality or lysosome damage. Moreover, the low viability of HUVEC-STs after NM treatment also showed that it is essential to evaluate the cytotoxicity index of various nanomaterials.

Interestingly, it was noted that neither the Alamar Blue test nor the neutral red assay showed a reduction in HUVEC-ST viability after treatment with PLA/EDTMP, PLGA/MDP, and PLA/MMT/TRA. As demonstrated by Helal-Neto et al.^28^, HUVEC exposition to non-loaded NP does not interfere with their viability. Controversially, in experiments carried out with bacterial strains, Guedes et al.^29^ have indicated high MDP toxicity. Our data have demonstrated how crucial it is to carry out different assays when studying the cytotoxic effect of NPs on normal cells^30^. Different HUVEC-ST sensitivity to the four different NP treatments may be associated with their various physicochemical properties. Thus, it was suggested that NPs with a positive charge bind to the negatively charged cell surface^31^. Consequently, these positively charged NPs can be imported into the cell more efficiently than negatively charged particles^32^. This hypothesis was confirmed by Huhn et al.^33^, who showed higher cytotoxicity of positively charged gold NPs in HUVECs compared to negatively charged gold NPs. The critical role of NP surface charge has also been proposed with a reference to NP absorption by serum proteins^34^. This phenomenon, known as the "corona effect", significantly increases the interactions between NPs and HUVECs and reduces toxicity^35^. DLS measurement results indicated that the examined NPs had a slightly positive charge and were marginally greater than 200 nm in size, with the exception of Pluronic F127 Ms, which had a diameter of 140 nm.

The size of NPs is an important factor in achieving an efficient desired effect, such as accumulation in tumor tissue or toxicity. Different toxicity profiles of PLA/MMT/TRA, PLA/EDTMP, PLGA/MDP, and Pluronic F127 Ms were observed not only in the viability assays, but also when HUVEC-ST proliferation rate was monitored following NP treatment for up to 72 h. Interestingly, the data obtained from the comet assay, one of the most popular assays used in toxicology studies to detect DNA damage^21^, revealed that all tested NPs possessed genotoxic properties, even though only Pluronic F127 Ms decreased HUVEC-ST viability. This high genotoxicity was confirmed by Mattos et al.^36^, who reported that 99mTc-MDP induced DNA strand breaks in rat blood cells.

The fatal consequences of DNA damage are often referred to cellular uptake of NPs. There are several endocytic mechanisms(e.g. clathrin mediated, clathrin-independent uptake) known with a membrane invagination diameter of less than 150 nm, whereas macropinocytosis is the most likely mechanism for uptake of NPs above this size^37^. Nevertheless, caveolae which are commonly implicated in trans-endothelial transport have diameters of 80 nm or less^38^, may be excluded here, as the possible mechanism of endocytosis, because they are smaller than all of four tested NPs. Interestingly, the most likely genotoxic properties of Pluronic F127 Ms can be partially related to the size of NPs and their reactivity. As particle size decreases, the particle unit of mass and overall surface area increases, because surface atoms have a tendency to possess high energy bonds. This larger surface area enhances catalytic activity, and consequently may lead to the oxidative stress and indirect induction of DNA lesions^39^.

On the other hand, cytotoxicity measurement and proliferation assay results were in contrast to the genotoxic profile of the investigated NPs. In support of these data, Calarco et al.^40^ have noted that micelles based on polyethyleneimine (PEI)-PLGA polymer induced DNA damage in HUVECs without a significant effect on cell viability, which showed that DNA damage can be a sensitive marker for NP-triggered toxicity.

It has been previously shown that several types of NPs, such as micelles based on polymers, silica NPs, or carbon nanotubes, can trigger DNA damage in HUVECs^41–43^. In agreement with these results, NPs in the present study induced DNA lesions after 24 and 48 h of treatment. When NP incubation was continued for up to 72 h, a decrease in DNA percentage was noted in the comet tail, suggesting that DNA repair had started.

There are many signalling pathways that are central to the maintenance of genome integrity. The role of ATM and ATR kinases has been well described in DNA repair, cell cycle regulation, and apoptosis^22^. There was a relationship between ATM and ATR gene mRNA level and NP toxicity in the present study. An increase in transcription of these two kinases suggested that ATM and ATR activity was rising due to the need to maintain genomic stability during exposure to DNA-damaging NPs. ATM and ATR kinases normally phosphorylate an overlapping set of DNA repair or checkpoint targets (p53, CHK2, NBS1, and BRCA1). By doing so, they ensure that the cells accurately repair DNA damage before DNA replication or cell division occurs^44^.

The results obtained in the present study clearly demonstrated that DNA lesions triggered by NPs can be interpreted as being due to the induction of DNA strand breaks and/or formation of alkali-labile sites, which can be transformed into strand breaks^23^. The breaks or alkali-labile sites may be the result of programmed cell death induced by the tested nanomaterials. Confirmation that the investigated NPs induced single or double-strand breaks was provided by the TUNEL assay and histone H2AX phosphorylation experiments. Many external factors such nanosubstances may lead to increased H2AX phosphorylation due to death-associated DNA fragmentation, especially long periods after drug treatment or in the case of agents that induce rapid apoptosis^45^. The characteristic oligonucleosomal fragmentation observed in the DNA ladder and the fraction of TUNEL-positive cells revealed that single-strand breaks were the highest during incubation. Interestingly, there were no evident differences between proapoptotic NP properties when considering Hoechst/PI double staining. The greatest population of necrotic cells that appeared after the NM treatment confirmed that these particles have very toxic properties. However, after analysing caspase-3 activity and its transcription after 24 h of incubation, the highest increase of these two parameters was observed in PLGA/MDP-treated samples. When NP incubation was prolonged to 48 h, the maximal caspase-3 activity was triggered by Pluronic F127 Ms, which was in agreement with PARP gene transcription measured when cells were treated with NPs for 24 h. Keeping in mind the cascade of molecular events where caspase-3 participates in PARP fragmentation, it can be concluded that Pluronic F127 Ms need more time to induce apoptosis in HUVEC-ST culture. Intriguingly, promising results for synergistic therapy between proteasome inhibitor PS-341 and ^153^Sm-EDTMP were revealed by Goel et al. Apoptosis induction (pro-caspase-3 and PARP cleavage) was observed in highly resistant myeloma cell lines after 24 h of combined treatment, even though neoplastic cell viability was 68%^46^.

There are many molecules like free radicals that act as second messengers and contribute to apoptosis induction. ROS production is also a hallmark of oxidative stress and an important mechanism of NP cytotoxicity. Numerous studies have established an important role for ROS in cell growth and division, e.g., low doses of ROS are necessary for cell proliferation, whereas high concentrations are inhibitory and apoptotic. In ECs, ROS function as second messengers to activate multiple intracellular proteins and enzymes, including the epidermal growth factor receptor, c-Src, p38 mitogen-activated protein kinase, Ras, and Akt/protein kinase B^47–49^. Direct induction of ROS in HUVEC-ST culture might lead to oxidative damage of DNA, proteins, or membranes. However, it must be noted that in addition to exogenous agents, endogenous cell components may also directly oxidize H_2_DCF-DA without the involvement of ROS as described for Cyt c^50,51^. Interestingly, it has been shown that the presence of antioxidant NAC can attenuate the adverse effects of Ag NPs and quantum dots (QDs) in HUVECs^52,53^. This finding was in contrast to the present results that showed the protective effect of NAC, which can be an extracellular source of amino acids essential for the synthesis of cytoplasmic scavengers. Perturbations of redox homeostasis are often related to mitochondrial dysfunction. As autonomous organelles, mitochondria are involved in ATP production. Damage to the mitochondrial oxidation chain in the endothelium results in an increase in oxidative stress parameters and has been implicated in cardiovascular disease^54^.

It has been previously shown that silica NPs and CdTe QDs provoke MMP collapse and release of mitochondrial Cyt c in HUVECs^41,52,55^. In the present study, the increase in Bcl2, AIF, and Cyt c gene transcription confirmed the hypothesis that long-term NP treatment induces mitochondria-dependent apoptosis, which is an intrinsic apoptosis pathway.

MMP disruption is characterized by the opening of permeability transition pores (PT) in the inner mitochondrial membrane. In the present study, an early decrease in MMPs 24 h after PLA/MMT/TRA and PLA/EDTMP treatment of HUVEC-ST cultures was observed. It seems that apoptosis induced by these NPs was due to disturbance in the generation of ROS necessary for cell proliferation. Interestingly, MMP hyperpolarisation, observed after 72 h of incubation with PLA/EDTMP, PLGA/MDP, or Pluronic F127 Ms, usually occurs before activation of caspases and phosphatidylserine externalization^56^, which may suggest secondary induction of apoptosis by these NPs^57^. Pluronic F127 Ms were 500-fold more toxic to HUVEC-STs than the remaining tested NPs PLA/MMT/TRA, PLA/EDTMP, and PLGA/MDP. Even though these three tested NPs did not decrease cell viability, they displayed a genotoxic effect and induced DNA damage. The formation of single and double-strand breaks was related to NP-triggered apoptosis induction. ROS production and perturbations of cellular redox homeostasis led to mitochondrial stress and contributed to the intrinsic apoptosis pathway.

Even though interactions of nanoparticles with e.g. cancer cells have been extensively studied, there is still not sufficient knowledge available about how the NPs behave in the body and how fast they may escape from blood and reach the target tissue. Firstly, it is essential to discover it could be interesting to study what is the effect of blood pressure, hemodynamic flow in blood vessels and velocity on interactions between the NPs and the vessel wall^58^. Besides, human blood is composed of plasma (containing salts, lipids, proteins, vitamins, hormones, and water) and importantly, a variety of immune cells (e.g., monocytes, neutrophils, B cells, dendritic cells (DCs), T cells, and (NK) cells^59^. For the future, understanding such important effect of blood pressure, hemodynamic flow in blood vessels as well as the velocity on interactions between the NPs and human blood vessels is our foundational goal.

In conclusion, the present study demonstrated that NPs potentially used in biomedicine displayed different levels of cytotoxicity in HUVEC-STs. The investigated NPs induced DNA damage, mainly oligonucleosomal DNA fragmentation, characteristic of programmed cell death. HUVEC-STs showed morphological markers of apoptosis following PLA/EDTMP, PLGA/MDP, and PLA//MMT/TRA treatment. Incubation with Pluronic F127 Ms caused typical cell necrosis and marked disruption of the cellular membrane. These NPs were able to generate ROS, which mediated a decrease in MMP and triggered the expression of genes involved in the intrinsic apoptosis pathway. These results demonstrated that the mechanism of action of the investigated NPs was highly type-dependent and their effect should be examined in other types of normal cells. Finally, the data underscored the necessity of a broad range of in vitro experiments in order to provide an in-depth prediction of NP cellular toxicity and their potential future directions.

## Methods

### Materials

Neutral red, resazurin, BCA assay, Tri Reagent, penicillin/streptomycin (Pen/Strep P4333), RNase A, Proteinase K, propidium iodide (PI), Hoechst 33258, *N*-acetyl cysteine (NAC), carbonyl cyanide m-chloro phenylhydrazone (FCCP), and bovine serum albumin (BSA) were purchased from Sigma-Aldrich Merck (Germany). Opti-MEM cell culture medium, fetal bovine serum (FBS), phosphate-buffered saline (PBS), caspase-3 assay, 5,5′,6,6′-tetrachloro-1,1′,3,3′-tetraethyl-benzimidazolcarbocyanine iodide (JC-1), dichlorodihydrofluorescein diacetate (H_2_DCF-DA), and TaqMan Reverse Transcription Reagents were obtained from Thermo Fisher Scientific. The SYBR-green PCR master mix was from Eurex (Gdansk, Poland), while the TUNEL assay and Human Phospho-Histone H2AX ELISA assay were bought from Biovision (California, USA) and R&D systems (London, UK), respectively. All reagents and solvents used in the production of NPs were acquired from Sigma-Aldrich, with the exception of MDP and EDTMP, which were acquired from ABX Advanced Biochemical Compounds, and TRASTUZUMAB, which was obtained from Roche Laboratories. All other chemicals were of reagent grade or better.

### Cell lines

HUVEC-STs (immortalized by transfection with both SV40 large/small T antigens and catalytic human telomerase subunit) was a kind gift from Dr. Claudine Kieda (University of Orleans, France) via a personal mediation by Prof. G. Bartosz (Department of Molecular Biophysics, University of Lodz, Poland). This cell line was established and characterized at the University of Rome Tor Vergata as described by Tentori et al.^60^. Cells were cultured in Opti-MEM medium supplemented with 3.5% fetal bovine serum, penicillin (100 U/mL), and streptomycin (100 mg/mL). Cells were grown at 37 °C in standard conditions (100% humidity, 37 °C, 95% normal air, and an atmosphere with 5% CO_2_). The cells were periodically tested for mycoplasma contamination and observed daily by microscopy for growth control. Logarithmic cell growth phase was maintained in all experiments.

### Formulation of investigated NPs

#### PLA/MMT/TRA NP production

A double emulsification technique was used to produce the PLA/MMT/TRA NPs. Briefly, 50 mg of PLA (Sigma-Aldrich) were dissolved in 2 mL of dichloromethane (Sigma-Aldrich) and added to 200 µL of PVA (Sigma-Aldrich) solution (0.1% w/v, and 50 mg of Trastuzumab (TRA, Herceptin, Roche Laboratories). This solution was sonicated (UP100H, Hielscher, Teltow, Germany) for two cycles of 30 s at 45 W. Then, 4 mL of aqueous PVA solution (0.7% w/v) and 0.42% clay (MMT; Montmorillonite; Sigma-Aldrich) were added and sonicated (UP100H, Hielscher, Teltow, Germany) for 60 s. The residual organic solvent was evaporated (Rotavapor R114, Buchi, Postfach, Switzerland) under reduced pressure for 1 h. Finally the NPs were recovered by ultra-centrifugation (20,000 rpm) at 25 °C for 20 min (Centrifugal Beckman Coulter J 25, Brea, California, USA). Physicochemical property measurements of freshly synthesized NPs were immediately performed^61^. Monoclonal antibody entrapment efficacy in PLA/MMT/TRA particles was measured using a BCA assay in parallel^62^.

#### PLGA/MDP NP production

Double emulsification technique was used for the production of PLGA/MDP NPs. A total of 1 mL of PVA (1% w/v; Sigma-Aldrich), 15 mg of MDP (ABX Advanced Biochemical Compounds, Germany), and 2 mL of PLGA (Sigma-Aldrich) solution (120 mg dissolved in 2 mL of dichloromethane) were sonicated (UP100H, Hielscher, Teltow, Germany) for 2 min at 55 W. Then, 50 mL of PVA (0.8% w/v; Sigma-Aldrich) were added and sonicated again for 5 min at 55 W resulting in a “water/oil/water” (W/O/W) solution. Finally the residual organic solvent was removed from the NPs by vacuum evaporation (Rotavapor R114, Buchi, Postfach, Switzerland) at 30 °C for 1 h and recovered by ultracentrifugation (15,000 rpm) at 25 °C for 20 min (Centrifugal Beckman Coulter J 25, Brea, California, USA). The NP properties were analysed just after synthesis by AMF microscopy^63^.

#### PLA/EDTMP NP production

For PLA/EDTMP NP production a double emulsion technique was used. Briefly, PLA (2.5% w/v; Sigma-Aldrich) was dissolved in 3 mL of methylene chloride (Sigma-Aldrich). A total of 200 µL of PVA (0.1% w/v; Sigma Aldrich) and EDTMP (4% w/v; ABX Advanced Biochemical Compounds, Germany) were added to this solution, followed by sonication (GEX 600 Sonicator Ultrasonic Processor) for 1 min at 0 °C and 55 W. Then, 4 mL of PVA (0.7% w/v) were added and sonicated again for 2 min (55 W) at 0 °C. The residual organic solvent was evaporated (Rotavapor R114, Buchi, Postfach, Switzerland) under reduced pressure for 20 min at 37 °C. Finally the NPs were recovered by ultra-centrifugation (20,000 rpm) at 25 °C for 10 min (Centrifugal Beckman Coulter J 25, Brea, CA, USA) and AMF images were taken if NPs were ready for experiments referred to as human endothelium cells^64^.

#### Pluronic F127 Ms production

The Pluronic F127 Ms was prepared using dispersed Pluronics F127 (Sigma-Aldrich) at a concentration of 12% (w/w) in water. Briefly, 100 mL of water were added to 12 mg of Pluronics F127 and gently stirred using a magnetic bar (Magnetic Stirrer, IKA, C-MAG HS-7) for 3 min and then processed for 3 min using an ultrasonic processor (UP100H, Hielscher, Power: 60%, Cycle: 1) in an ice bath at 10 °C. After the synthesis, transmission electron microscopy (TEM) analysis of the obtained Pluronic F127 Ms was carried out ^65^.

### Characterization of investigated NPs

To demonstrate the breadth and variety of nanosubstance effects in human ECs, four different NPs were used in cancer therapy, theranostics, and cosmetology (Table 1, Fig. 1). NP zeta potential (ζ), PDI, and size (hydrodynamic diameter) were measured in 0.01 M phosphate buffer at pH 7.4 (PBS) and 25 °C using dynamic light scattering (DLS) and a Zetasizer Nano ZS (Malvern Instruments, UK). The NP suspensions were diluted to 1 mg/mL with PBS before analysis and the measurements were performed in triplicate maintaining the device in automatic mode. The purification steps were performed before the in vitro experiments. Sterilizing filtration was used for NPs sized < 220 nm, whereas for particles with greater sizes, NPs were lyophilized and then resuspended in sterile water. The final step referred to the analyzed NP description of entrapment efficacy (% of encapsulated drug) calculation described previously^66–68^.

### Cytotoxicity assays

Cytotoxicity was estimated by performing the neutral red assay (using a dye that passes through intact plasma membranes of viable cells and is accumulated in lysosomes), Alamar Blue assay (by measuring resazurin oxidation), and observing resulting cell morphology changes under a microscope.

### Neutral red assay

Cells in 96-well plates were treated with PLAMMT/TRA, PLA/EDTMP, PLGA/MDP (concentration range 0–500 μg/mL), or Pluronic F127 Ms (concentration range 0–1 μg/mL) for 72 h. Thereafter, the medium was removed and replaced with a neutral red solution (0.5 mg/mL). The incubation was continued for a further 3 h under cell growth conditions. At the end of incubation, neural red imported into fully functionalized live cell lysosomes was dissolved in the extractant solution (51% H_2_O, 48% ethanol, and 1% acetic acid). The absorbance was measured at 550 nm (analytical wavelength) and 620 nm (reference wavelength) with a microplate reader (Awareness Technology Inc., USA).

### Alamar blue assay

Cells (2 × 10^3^) in 100 μL of culture medium per well were seeded into black 96-well microtiter plates with flat-bottomed wells 24 h before the experiment and then exposed in triplicate to different concentrations of PLA/MMT/TRA, PLA/EDTMP, PLGA/MDP, or Pluronic F127 Ms for 72 h (5% CO_2_, 37 °C, 100% humidity). Cell medium was aspirated and replaced with 100 μL of resazurin solution at the end of incubation. Incubation was continued for a further 3 h and then fluorescence was measured using a microplate reader (Fluoroskan Ascent FL, Sweden) with excitation and emission wavelengths of 530 nm and 570 nm, respectively.

### Cell morphology

Cell morphology alterations were analysed after 24-h exposure to NPs. Images were acquired using an inverted microscope (Olympus IX70, Japan) equipped with a 20 × objective and a Digital Sight camera (Olympus, Tokyo, Japan).

### Growth inhibition assay

The cells used to determine the growth rate were seeded at a density of 0.5 × 10^5^ cells per sample and cultured for up to four days. The number of viable cells was evaluated every 24 h using the trypan blue exclusion method.

### Comet assay

To measure DNA damage, the comet assay was performed under alkaline conditions according to the slightly modified procedure described by Singh et al.^69^. HUVEC-STs were treated with NPs for up to 72 h (24, 48, and 72 h), and cells treated with 10 μM hydrogen peroxide for 10 min at 4 °C were used as a positive control. After electrophoresis (29 V, 30 mA, 4 °C) and DAPI staining, the slides were analysed at 400 × magnification using an Olympus fluorescence microscope (Olympus, Tokyo, Japan) and attached to an Olympus video camera (Olympus, Tokyo, Japan) equipped with a UV filter. Fifty cell images were selected from each sample and each experiment was repeated three times. The samples were analysed with a free version of the *CASP* software^70^.

### RNA extraction and quantitative real-time polymerase chain reaction (qRT-PCR)

ATM, ATR, AIF, Bax, Bcl-2, Casp-3, H2AX, and PARP (the primer sequences are listed in Table 2) mRNA expression levels were measured by qRT-PCR as described previously^71^. Total RNA was extracted using the TRI Reagent (Sigma-Aldrich, USA) according to the manufacturer instructions, and reverse transcription to cDNA was carried out using the TaqMan Reverse Transcription Reagents (Thermo Scientific, USA). The qRT-PCR was performed with the SYBR-green PCR master mix (EURx, Gdansk, Poland) in an Eco Real-Time PCR System (Illumina, USA). The cycling conditions were 94 °C for 4 min, followed by 40 cycles at 94 °C for 15 s, 60 °C for 25 s, and 72 °C for 25 s. The housekeeping gene hypoxanthine phosphoribosyltransferase 1 (HPRT1) served as an internal control and Eco Real-Time PCR Relative Quantification Software (Illumina, USA) was used for quantification. Data (ΔCt values) were transformed into relative copy number values (the number of mRNA copies of the examined genes per housekeeping gene index), calculated as the average Ct value of the HPRT1 housekeeping gene, and standardized to the level of mRNA transcripts in untreated cells, taken as 1.

**Table 2:** Primer sequences used for RT-PCR.

Gene	Strand	Sequence 5′–> 3′
Hypoxanthine–guanine phosphoribosyltransferase (HPRT1)	Forward	TGACACTGGCAAAACAATGCA
	Reverse	GGTCCTTTTCACCAGCAAGCT
Bcl2-like protein 4 (Bax)	Forward	GTTTCATCCAGGATCGAGCAG
	Reverse	CATCTTCTTCCAGATGGTGA
B-cell lymphoma 2 (Bcl2)	Forward	AGGAAGTGAACATTTCGGTGAC
	Reverse	GCTCAGTTCCAGGACCAGG
Cytochrome complex (Cyt c)	Forward	AGGCCCCTGGATACTCTTACACAG
	Reverse	TCAGTGTATCCTCTCCCCAGATG
H2A histone family member X (H2AX)	Forward	ACGAGGAGCTCAACAAGCT
	Reverse	GTGGCGCTG GTCTTCTTG
ATM serine/threonine kinase (ATM)	Forward	GCAGCTGGAAGAAGCACAA
	Reverse	TTTTAGGCTGGGATTGTTCG
Ataxia telangiectasia and Rad3-related protein (ATR)	Forward	TGTAGAGAGATGGAGACCAACG
	Reverse	GACCAATCGGTTGACTTCTGA
Poly (ADP-ribose) polymerase (PARP)	Forward	GTGTGGGAAGACCAAAGGAA
	Reverse	TTCAAGAGCTCCCATGTTCA
Cytochrome c (Cyt c)	Forward	AGGCCCCTGGATACTCTTACACAG
	Reverse	TCAGTGTATCCTCTCCCCAGATG
Apoptosis inducing factor (AIF)	Forward	ATGGAGTTTGACCTTAATGG
	Reverse	GCAACTCAGAGATAGCTTTG

### TUNEL assay

To evaluate whether DNA damage induced by NPs (100 μg/mL of PLA/MMT/TRA, PLA/EDTMP, PLGA/MDP, and 0.025 μg/mL of Pluronic F127 Ms) was related to apoptosis induction, TUNEL (TdT-mediated dUTP Nick-End Labelling) assay was performed following previously described procedure^72^. This method revealed the early apoptosis stage by labelling 3′OH ends of single- and double-stranded DNA fragments with fluorescein-12-dUTP. After 24-, 48-, and 72-h incubation with NPs, particle-treated and control cells were processed according to the TUNEL assay DNA-fragmentation kit protocol supplied by the manufacturer (Biovision, USA). The green fluorescence of fluorescein-12-dUTP incorporated at the DNA damage sites was detected by flow cytometry (BD Biosciences, USA). The dye was excited using a 488-nm Ar laser and detected with the FL1 (545 nm) detector on an LSR II Flow Cytometer. At least 10,000 cells were recorded for each readout. The number of TUNEL-positive cells was calculated as a percentage of the total number of cells in the sample.

### Oligonucleosomal DNA fragmentation

To verify whether NPs induced internucleosomal DNA fragmentation in HUVEC-STs, the procedure described by Gong et al.^73^ was followed with some slight modifications. Briefly, after 72 h of incubation with the NPs, DNA was extracted with 0.2 M phosphate-citrate buffer at pH 7.8 and 37 °C for 30 min. Subsequently, the samples were centrifuged at 1500×*g* for 10 min and the supernatant containing low molecular weight DNA (LMW DNA) was treated with RNase A (1 mg/mL) and Proteinase K (1 mg/mL) for 30 min at 37 °C. DNA samples were fractioned by electrophoresis on 1.8% agarose gel stained with ethidium bromide (0.5 mg/mL) for 2 h at 100 V in TBE buffer (100 mM Tris HCl, 0.1 M boric acid, and 1.5 mM EDTA, pH 8.0). The gels were visualized under UV illumination using an In Genius Bio Imaging System (Syngene International Limited, India) in the Department of Molecular Genetics, University of Lodz.

### HISTONE H2AX phosphorylation

HISTONE H2AX phosphorylation was measured using the Human Phospho-Histone H2AX DuoSet IC ELISA kit according to the manufacturer instructions (R&D Systems, UK). A total of 2 × 10^6^ cells were incubated for 24, 48, and 72 h with NPs. Following incubation, the cells were lysed with lysis buffer supplied by the assay manufacturer. Clarified cell extracts were added to triplicate wells to determine the cellular level of phospho-histone H2AX and total histone concentration. The resulting fluorescence was measured on a Fluoroskan Ascent plate reader (Fluoroskan Ascent FL, Sweden) with filter pairs of 540/600 nm and 360/450 nm. The results were presented as a fluorescence ratio measured at 540/600 nm to that measured at 360/450 nm (phosphorylated form to total Histone H2AX concentration) relative to the control cells.

### Apoptosis and necrosis detection

The number of cells in various stages of cell death were analysed by double staining with Hoechst 33258 and PI as described previously^72^. Cells were cultured with various concentrations of NPs (100 μg/mL of PLA/MMT/TRA, PLA/EDTMP, PLGA/MDP, and 0.025 μg/mL of Pluronic F127 Ms) for 24, 48, and 72 h. At certain time points, the cells were removed from culture dishes by trypsinization, centrifuged, suspended in HBSS at a final concentration of 1 × 10^6^ cells/mL, and then stained for 5 min at 37 °C with Hoechst 33,258 (0.13 mM) and PI (0.23 mM). The cell suspension (50 μL) was placed on a microscope slide and at least 300 cells/sample were counted under a fluorescence microscope (Olympus IX70, Japan). The cells were divided into four categories as follows: living cells (pale-blue fluorescence), early apoptotic cells (intensive bright-blue fluorescence), late apoptotic cells (blue-violet fluorescence), and necrotic cells (red fluorescence). The analysis was repeated three times and the results were presented as means ± SD, n = 3.

### Measurement of caspase-3 activity

Caspase-3 activity was evaluated using the fluorometric assay kit (Caspase-3 Activity Assay, Thermo Fisher Scientific, USA) according to the manufacturer instructions^72^. HUVEC-STs were incubated with NPs (100 μg/mL of PLA/MMT/TRA, PLA/EDTMP, PLGA/MDP, and 0.025 μg/mL of Pluronic F127 Ms) for 24, 48, and 72 h and lysed thereafter. Lysates were mixed in the reaction buffer and incubated with the caspase-3 substrate (Z-DVED-R110). Fluorescence intensity of the released product was measured using a Fluoroskan Ascent FL plate reader (Labsystem, Sweden) with the excitation and emission wavelengths of 496 nm and 520 nm, respectively. The obtained results were normalized to the protein content estimated using the Lowry method (Lowry, Rosebrough et al. 1951) from the standard curve for bovine serum albumin.

### ROS formation assay

To measure intracellular ROS formation, fluorescent probe dichlorodihydrofluorescein diacetate (H_2_DCF-DA) was used. H_2_DCF-DA was diffused into cells and deacetylated by cellular esterase to non-fluorescent 2′,7′-dichlorodihydrofluorescein, which was rapidly oxidized by ROS to highly fluorescent 2′,7′-dichlorofluorescein. Fluorescence intensity is proportional to ROS levels within cell cytosol. Briefly, cells were seeded in 96-well plates and incubated with NPs for 24, 48, and 72 h. In some experiments, 1-h cell pre-incubation with 3 mM antioxidant (N-acetyl cysteine, NAC) was performed before the NPs were added and incubation was continued for the required period of time under the same conditions. Subsequently, the cells were incubated with 5 μM H_2_DCF-DA at 37 °C for 30 min^74^ and ROS fluorescence (DCF) was measured using a Fluoroskan Ascent FL microplate reader (Labsystems, Sweden).

In addition, DNA content was measured in NP-treated samples in order to avoid measurement errors in fluorescence intensity caused by cell detachment in the well^75^. After measuring H_2_DCFDA fluorescence, the probes were removed by gentle aspiration, 100 μL of deionized water were added to the appropriate wells, and the microplate with cells was frozen at − 70 °C. Before measurement, the microplate with cells was thawed at room temperature and RNA was digested with RNAse. Then, 100 μL of 5 μM PI were added, the plate was incubated for 15 min at room temperature in the dark, shaken, and the fluorescence was read at 535/617 nm by a Fluoroskan Ascent FL microplate reader (Labsystems, Sweden).

### Mitochondrial membrane potential (ΔΨm)

HUVEC-STs were seeded into 96-well microplates. After 24 h, various concentrations of investigated NPs (100 μg/mL of PLA/MMT/TRA, PLA/EDTMP, PLGA/MDP, and 0.025 μg/mL of Pluronic F127 Ms) or a concentration range of 0.01–5 μM for FCCP, an uncoupling mitochondrial agent (positive control), were added to the wells. The cells were incubated with the analyzed NPs or FCCP for 24, 48, and 72 h. The medium was removed at the end of the treatment, and the cells were incubated in the dark with 5 μM JC-1 in HBSS for 30 min at 37 °C. JC-1 is a fluorescent carbocyanine dye, which accumulates in the mitochondrial membrane in two forms (monomers or dimers), depending on mitochondrial membrane potential. JC-1 monomers show maximum fluorescence excitation and emission at 485 and 538 nm wavelengths, respectively^76^. The fluorescence of both JC-1 monomers and dimers was measured on a Fluoroskan Ascent FL microplate reader (Labsystems, Sweden) using filter pairs of 530/590 nm (dimers) and 485/538 nm (monomers).

### Statistical analysis

The data were presented as the mean ± SD from three independent experiments and estimated for normal distribution with the Shapiro–Wilk test. The sample size was calculated for type I and type II statistical errors of 0.05 and 0.01, respectively. Subsequently, homogeneity variance was verified with Levene's test. Evaluation of statistically significant differences between control and NP-treated samples was performed using univariate analysis of variance and Tukey's test with a post hoc analysis as described previously^71^. A p-value of 0.05 was considered significant. Statistical analysis was performed using STATISTICA.PL software v.12 (StatSoft, Poland). In addition the viability curves were prepared using the GraphPad Prism 5.0 software (GraphPad Inc., USA).

### Consent for publication

The manuscript has been read and approved by all the authors, the requirements for authorship have been met, and each author believes the manuscript represents honest work.

## Supplementary information

Supplementary information.

## Data Availability

All data generated or analysed during this study are included in this published article and its additional file.
