# Supplementary material for: Disturbance of cellular homeostasis as a molecular risk evaluation of human endothelial cells exposed to nanoparticles
Source: Sci Rep. 2021 Feb 15;11:3849. doi: 10.1038/s41598-021-83291-0 (PMC7884700; doi:10.1038/s41598-021-83291-0)
Supplement: Supplementary file 1 — Supplementary information. [file 41598_2021_83291_MOESM1_ESM.docx]

**Disturbance of cellular homeostasis as a molecular risk evaluation of human endothelial cells exposed to nanoparticles**

Paulina Wigner^1,#^ Krzysztof Zielinski^1,#^, Sylwia Michlewska^2^, Paulina Danielska^1^, Agnieszka Marczak^1^, Eduardo Junior Ricci^3^, Ralph Santos - Oliveira^4,5^ & Marzena Szwed^1,*^

^1^Department of Medical Biophysics, Institute of Biophysics, Faculty of Biology and Environmental Protection, University of Lodz, Lodz, Poland

^2^ Laboratory of Microscopic Imaging and Specialized Biological Techniques, Faculty of Biology and Environmental Protection, University of Lodz, Lodz, Poland

^3^Laboratory of Nanodrugs, Faculty of Pharmacy, Rio de Janeiro Federal University, Rio de Janeiro, Brazil

^4^Laboratory of Nanoradiopharmaceuticals, Zona Oste State University, Research Group of Nano-Radiopharmaceuticals and Novel Radiopharmaceuticals, Rio de Janeiro, Brazil

^5^Laboratory of Nanoradiopharmacy and Synthesis of Novel Radiopharmaceuticals, Nuclear Engineering Institute, Brazilian Nuclear Energy Commission, Rio de Janeiro, Brazil

^#^Contributed equally

^*^Corresponding author

^*^Correspondence:

Dr. Marzena Szwed

E-mail: marzena.szwed@biol.uni.lodz.pl

Contact: +486354481/ +48506612428

| **Author order** | **Author name** | **Affiliation** | **E-mail address** | **OCRID NUMBER** |
| --- | --- | --- | --- | --- |
| ^1,#^Contributed equally | Paulina Wigner # | Department of Medical Biophysics, Institute of Biophysics, University of Lodz, Lodz, Poland | paulina.wigner@biol.uni.lodz.pl |  |
| ^1.#^Contributed equally | Krzysztof# Zielinski | Department of Medical Biophysics, Institute of Biophysics, University of Lodz, Lodz, Poland | [kris1400@gmail.com](mailto:kris1400@gmail.com) |  |
| ^2^ | Sylwia Michlewska | Laboratory of Microscopic Imaging and Specialized Biological Techniques, University of Lodz, Lodz, Poland | sylwia.michlewska@biol.uni.lodz.pl | 0000-0002-8952-469X |
| ^3^ | Paulina Danielska | Department of Medical Biophysics, Institute of Biophysics, University of Lodz, Lodz, Poland | paulina.danielska.pd@gmail.com |  |
| ^4^ | Agnieszka Marczak | Department of Medical Biophysics, Institute of Biophysics, University of Lodz, Lodz, Poland | agnieszka.marczak@biol.uni.lodz.pl | 0000-0003-3439-9032 |
| ^5^ | Eduardo Junior Ricci | Laboratory of Nanodrugs, Faculty of Pharmacy, Rio de Janeiro Federal University, Rio de Janeiro, Brazil | ricci@pharma.ufrj.br. | 0000-0002-2550-696X |
| ^6^ | Ralph Santos-Oliveira | Laboratory of Nanoradiopharmaceuticals, Zona Oste State University, Research Group of Nano-Radiopharmaceuticals and Novel Radiopharmaceuticals, Rio de Janeiro, Brazil  Laboratory of Nanoradiopharmacy and Synthesis of Novel Radiopharmaceuticals, Nuclear Engineering Institute, Brazilian Nuclear Energy Commission, Rio de Janeiro, Brazil | presidenciaradiofarmacia@gmail.com | 0000-0002-7221-6404 |
| ^7^ Corresponding author | Marzena Szwed* | Department of Medical Biophysics, Institute of Biophysics, University of Lodz, Lodz, Poland | marzena.szwed@biol.uni.lodz.pl | 0000-0002-2102-3741 |

| **1 2 3 4 5 6** |
| --- |
| **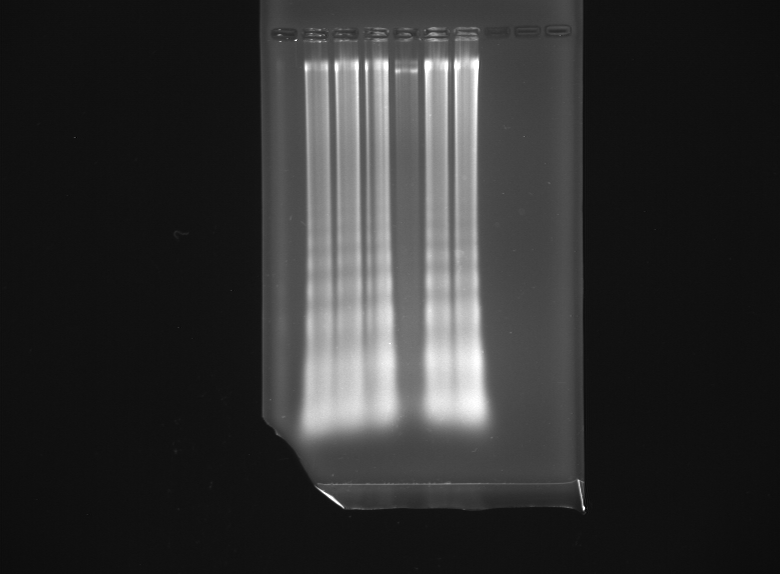** |
|  |

**Figure S1** DNA fragmentation induced by NPs detected by agarose gel electrophoresis of DNA isolated from human endothelial cells. HUVEC-STs were treated for up to 72 h with 100 μg/mL of PLA/EDTMP, PLGA/MDP, and PLA/MMT/TRA or 0.025 μg/mL of NMs; 1 - PLA/MMT/TRA, 2 - PLA/EDTMP, 3 - PLGA/MDP, 4 – Control, 5 – NMs, 6 – NMs modified with chitosan – not shown in the main manuscript.
